# The effects of *Bacillus coagulans* MTCC 5856 on functional gas and bloating in adults: A randomized, double-blind, placebo-controlled study

**DOI:** 10.1097/MD.0000000000033109

**Published:** 2023-03-03

**Authors:** Muhammed Majeed, Kalyanam Nagabhushanam, Shaji Paulose, Sivakumar Arumugam, Lakshmi Mundkur

**Affiliations:** a Sami-Sabinsa Group Limited, Karnataka, India; b Sabinsa Corporation, NJ.

**Keywords:** *Bacillus coagulans* MTCC 5856, bloating, gas, GSRS, probiotic

## Abstract

Gut microbiome dysbiosis is a major cause of abdominal gas, bloating, and distension. *Bacillus coagulans* MTCC 5856 (LactoSpore) is a spore-forming, thermostable, lactic acid-producing probiotic that has numerous health benefits. We evaluated the effect of Lacto Spore on improving the clinical symptoms of functional gas and bloating in healthy adults.

**Methods:**

Multicenter, randomized, double-blind, placebo-controlled study at hospitals in southern India. Seventy adults with functional gas and bloating with a gastrointestinal symptom rating scale (GSRS) indigestion score ≥ 5 were randomized to receive *B coagulans* MTCC 5856 (2 billion spores/day, N = 35) or placebo (N = 35) for 4 weeks. Changes in the GSRS-Indigestion subscale score for gas and bloating and global evaluation of patient’s scores from screening to the final visit were the primary outcomes. The secondary outcomes were Bristol stool analysis, brain fog questionnaire, changes in other GSRS subscales, and safety.

**Results:**

Two participants from each group withdrew from the study and 66 participants (n = 33 in each group) completed the study. The GSRS indigestion scores changed significantly (*P* < .001) in the probiotic group (8.91–3.06; *P* < .001) compared to the placebo (9.42–8.43; *P* = .11). The median global evaluation of patient’s scores was significantly better (*P* < .001) in the probiotic group (3.0–9.0) than in the placebo group (3.0–4.0) at the end of the study. The cumulative GSRS score, excluding the indigestion subscale, decreased from 27.82 to 4.42% (*P* < .001) in the probiotic group and 29.12 to 19.33% (*P* < .001) in the placebo group. The Bristol stool type improved to normal in both the groups. No adverse events or significant changes were observed in clinical parameters throughout the trial period.

**Conclusions:**

*Bacillus coagulans* MTCC 5856 may be a potential supplement to reduce gastrointestinal symptoms in adults with abdominal gas and distension.

## 1. Introduction

Functional bowel disorders (FBD) are highly prevalent, affecting all facets of society globally. They reduce people’s quality of life and have a negative impact on the global health care system.^[1]^ FBDs are classified into irritable bowel syndrome (IBS), functional abdominal bloating/distention, functional diarrhea, functional constipation, and unspecified FBD. Bloating is a perception of retained gas, discomfort, fullness, and abdominal pressure. At the same time, distensions increase abdominal circumference.^[2]^ Functional abdominal bloating is defined as a feeling of abdominal fullness in the absence of any other functional gastrointestinal disorder. The diagnostic criteria include the presence of abdominal distension, bloating, and a feeling of abdominal fullness for 12 weeks, (not necessarily continuous) but with insufficient criteria for the diagnosis of irritable bowel syndrome, functional dyspepsia, or other functional disorders.^[3]^ It is generally absent in the morning and worsens through the day. About 16% to 30 % of the general population suffers from functional abdominal bloating and distension.^[4,5]^

Although the etiology of bloating and distension is not well understood, small intestinal bacterial overgrowth (SIBO), gut microbiota alterations, intolerance to food/carbohydrates, visceral hypersensitivity, abnormal intestinal gas transit, and gas evacuation are a few common triggers of bloating.^[1,6]^ Maldigestion of carbohydrates and excess growth of bacteria induce gas production, causing stretching and distension of the intestinal tract.

Abdominal bloating can also result from IBS, functional dyspepsia, constipation, and pelvic floor dysfunction. Diet modification, antibiotics, prokinetic agents, probiotics, antispasmodics, and neuromodulators are therapeutic options for bloating and distension.^[5]^ These therapies are effective and provide symptomatic relief, but they do not address the natural history of the problem and are often associated with adverse effects.^[7]^

Probiotics are defined as “live microorganisms that, when administered in adequate amounts, confer a health benefit on the host” ‘.^[7]^ Probiotic supplementation is effective across a diverse spectrum of gastrointestinal (GI) disorders, such as SIBO, antibiotic-associated and infectious diarrhea, inflammatory bowel disease, and IBS.^[8–10]^

The spores of *Bacillus coagulans* have gained attention due to their resistance to heat and hostile gastrointestinal conditions.^[11]^ It was initially isolated from spoilt milk by Hammer in 1915.^[12]^ Since then, several strains have been reported from different sources. *B coagulans* Ganeden BC30, SNZ 1969, Unique IS-2, Thorne, PTA-6086, PTA-11748, SANK 70258, and PROBACI are some of the strains apart from MTCC5856.^[11,13]^ The spores pass through the stomach, withstanding the gastric juices and bile, and start germinating in the duodenum. They proliferate in the nutrient-rich environment of the small intestine and sporulate again in the lower part of the colon before excretion.^[14,15]^ Most importantly, despite producing acid, *B coagulans* does not produce gas from glucose fermentation.^[13]^ Additionally, different strains of *B coagulans* are reported to have antimicrobial activity against bacterial pathogens.^[16–18]^

The probiotic strains belonging to the same species are known to vary in their properties due to differences in their genotypic and phenotypic characteristics,^[19]^ and show strain and disease-specific biological activity.^[20]^ *B coagulans* species have been shown to produce digestive enzymes, increase nutrient absorption, and help digestion and other gastrointestinal diseases.^[21–23]^ *B coagulans* strains can also produce short-chain fatty acids, improve the intestinal environment, promote healthy bowel movement, and enhance the health of gut cells.^[24,25]^ *B coagulans* MTCC 5856 is a non-genetically modified organism with US FDA-reviewed generally recognized as safe status.^[26]^ It has been available on the market for nearly 3 decades and is safe for humans at the dose of 2 billion spores per day.^[27–30]^It has gastrointestinal motility inhibiting effects, displays antidiarrheal activity, and reduces vomiting, diarrhea, abdominal pain, and stool frequency in IBS patients.^[29,30]^

The present study evaluated the efficacy of *B coagulans* MTCC 5856 at a dose of 2 billion CFU/day to manage functional gas and bloating symptoms in patients with abdominal discomfort, gas, bloating, and distension in the absence of other gastrointestinal disorders.

## 2. Materials and methods

### 2.1. Materials

*Bacillus coagulans* MTCC 5856 (LactoSpore^®^) was provided by the Sami-Sabinsa Group. Each tablet contained 2 × 10^9^ spores, microcrystalline cellulose, Aerosil, Magnesium Stearate, and Sodium Starch Glycolate. In placebo tablets, the weight of *B coagulans* MTCC 5856 spores was replaced with maltodextrin, while the other ingredients were precisely similar. The average weight of both tablets was 410 mg.

### 2.2. Gastrointestinal symptom rating scale

The gastrointestinal symptom rating scale (GSRS) is a 15-item questionnaire to assess the common symptoms associated with GI disorders. GSRS utilizes a 7-level Likert scale (0–6) depending on the intensity and frequency of GI symptoms experienced during the previous week. It is divided into 5 subscales. Abdominal pain, gastric hunger pain, and nausea scores are grouped under “Abdominal Pain.” Reflux, heartburn, and regurgitation scores are included in the Reflux or “Dyspepsia syndrome.” Abdominal distension, borborygmi, burping, and flatulence are included under the “Indigestion syndrome.” “Diarrhea” is evaluated based on the increased frequency of evacuation, loose stools, urgent need to defecate, and constipation by difficulty in defecating.^[31,32]^ Each subscale score ranges from 0 to 6, with higher scores reflecting greater discomfort. The indigestion subscale included 3 questions related to feeling bloated, bothered by burping and passing gas or flatus. The cumulative score of these questions (>5) were considered for inclusion.

### 2.3. Ethics and study design

The trial was conducted as a prospective, randomized, double-blind, placebo-controlled at 2 investigative sites in Bangalore (MS Ramaiah Medical College and Hospitals and Santosh Hospital) between August 2019 and March 2021. The Institutional Ethics Committees of both sites approved the study. Written informed consent was taken from all the subjects before enrollment in the study. The trial was conducted per the Declaration of Helsinki, the International Conference on Harmonization Guidelines for Good Clinical Practice, and applicable local regulations and was registered prospectively with the clinical trial registry of India with the registration number clinical trial registry of India/2019/06/019617.

### 2.4. Sample size

The study statistician calculated the sample size based on earlier studies using a power of 80% and an alpha significance of 0.05, and correlation of 0.32, the required total sample size was calculated to be 60 for evaluation. Considering a 15% drop out rate, 70 participants were recruited and randomized in a 1:1 ratio between *B coagulans* and placebo.^[33]^

### 2.5. Inclusion and exclusion criteria

The study included adult (18–65-year-old) male and female participants experiencing abdominal discomfort, gas, bloating, and distension symptoms. The enrolled subjects fulfilled Rome IV C4 diagnostic criteria for functional abdominal bloating/distension. Rome IV C4 diagnostic criteria consist of recurrent bloating and/or distention occurring at least 1 day per week and a predominance of abdominal bloating and distention over other symptoms in the absence of IBS, functional constipation, functional diarrhea, or postprandial distress syndrome. As per the Rome IV criteria, these symptoms should be present for at least 6 months before diagnosis, with persisting symptoms for the last 3 months.^[1]^ The GSRS, indigestion subscore for all enrolled subjects was >5, suggesting mild to moderate symptoms. Other inclusion criteria included a willingness to complete subject diaries and questionnaires, abstaining from prebiotic and probiotic food supplements, vitamins, proteins, and minerals supplements, laxatives, a high fiber diet, and dairy products during the study.

Exclusion criteria included indications of functional dyspepsia or other functional gastrointestinal disorders; active psychiatric conditions; and consumption of supplements or medications that would interfere with the gut’s natural microbiota, such as antibiotics, within the last 21 days before screening. In addition, subjects with gastrointestinal disorders or other digestive problems such as Crohn disease, short bowel, ulcerative colitis confirmed by fecal calprotectin negative test, constipation, and lactose intolerance were also excluded. Subjects who used gastrointestinal medications to control gut function, such as antispasmodics, prokinetic agents, probiotics, prebiotics, or laxatives, were excluded from the study.

### 2.6. Randomization, blinding, and intervention

Subjects were randomized using computer-generated random allocation software (STATA Software version 16.0, StataCorp LLC, College Station, TX). An alphabetic code was generated for both the *B coagulans* and placebo to improve the blindness of the study and concealment of allocations following the block randomization method. The randomization sequence was prepared by a statistician independent of the sponsoring organization, who was not involved in the conduct or reporting of the study. All the study staff, investigators, and subjects were blinded throughout the study. The participants were instructed to consume *B coagulans* MTCC 5856 tablets or an identical placebo orally once daily, 30 minutes before food in the morning, for 4 weeks. Treatment compliance was monitored by recording the number of tablets dispensed and those returned at each visit to the case report form.

### 2.7. Outcome measures

The participants completed all the 15 questions in the GSRS questionnaire. Three questions related to feeling bloated, bothered by burping and passing gas or flatus were considered as indigestion subscale. The primary outcome was the change in the cumulative indigestion score from screening to end of the study. Change in the global evaluation of patients’ scores, (a 10-point visual analogue scale where 1 is bad and 10 is good) from screening to the final visit was another primary outcome measure. The scores were assessed at the screening visit (visit 1), visit 3 (day 15), and the final visit (day 30).

The secondary outcomes were a change in other subscale scores in the GSRS questionnaire, excluding the indigestion score from screening to the final visit; a change in Bristol stool analysis from screening to the final visit; a change in brain fog questionnaire score from screening to the final visit; and safety by assessing the adverse events. The Bristol stool chart and the Brain Fog questionnaire were assessed at the screening and final visits, while adverse events were monitored throughout the study. As a part of safety, laboratory parameters like hematology, renal function test, liver function test, and urine analysis were performed at screening and the final visit.

### 2.8. Statistical analysis

All the statistical analysis was performed by STATA Software version 16.0. The GSRS Indigestion subscale score, global evaluation of patient scores, and other cumulative subscale scores in the GSRS questionnaire were represented as continuous variables.

A comparative analysis was performed for normally distributed data within the group, and the results were presented as mean, standard deviation/standard error, and *P* value. For nonnormally distributed data within the comparative group, analysis was performed using the Wilcoxon Signed-Rank test. The results were presented as median, range, and *P* value. An unpaired *t* test/Mann-Whitney test was performed for the comparative analysis between treatment groups. The level of statistical significance for each test is defined as *P* < .05. Other secondary endpoints, Bristol stool analysis, brain fog questionnaire, and the occurrence of adverse events, were presented as categorical variables. A descriptive comparison was provided to differentiate the treatment effects between the treatment groups and within treatment groups.

## 3. Results

### 3.1. Demographic characteristics

**Figure 1.**
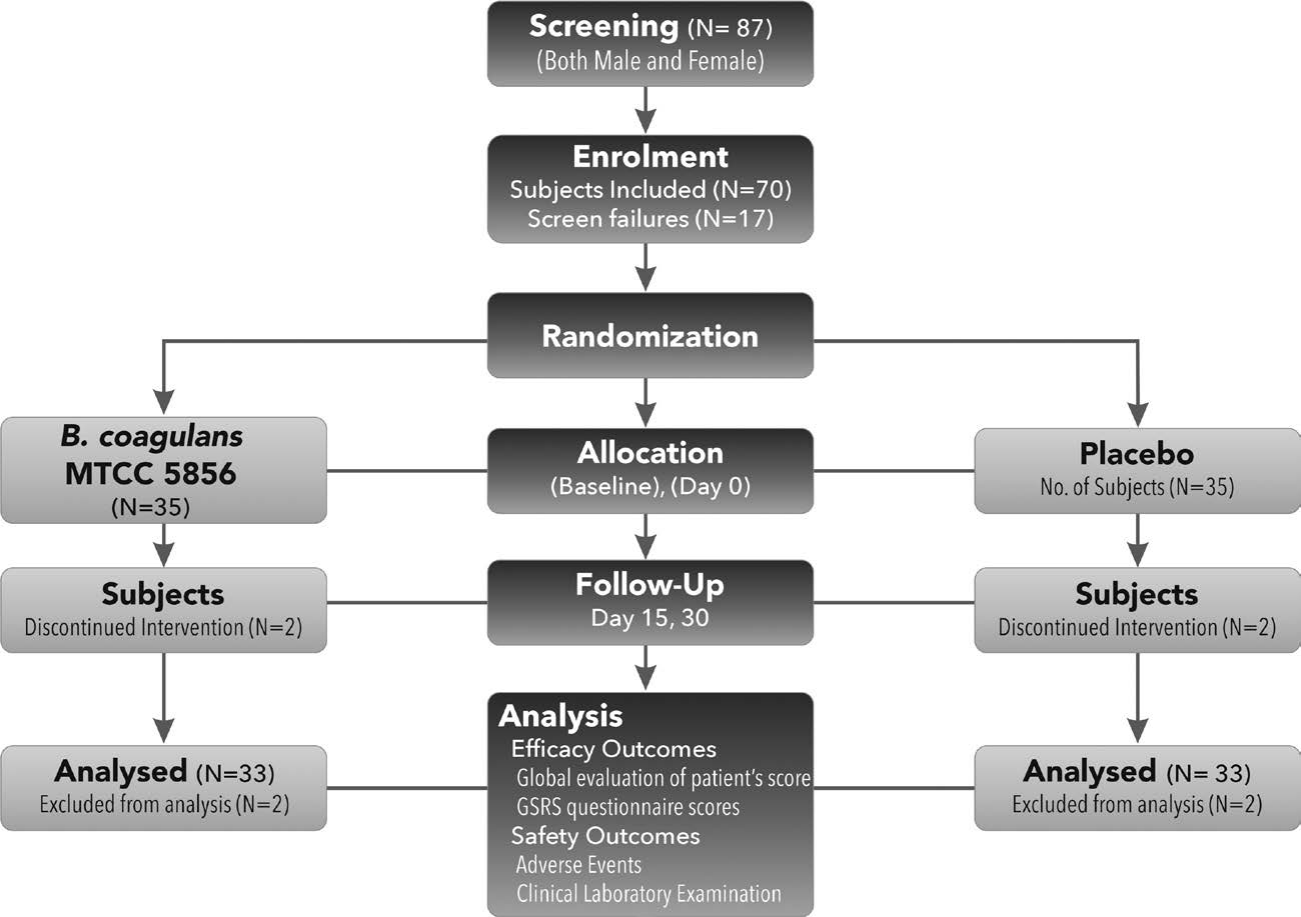
CONSORT flow diagram.

A total of 87 subjects were screened, at the participating hospitals for the presence of functional abdominal bloating as per the inclusion/exclusion criteria, and 70 (29 males and 41 females) were enrolled and randomized to the *B coagulans* and placebo groups (N = 35 each). Four subjects discontinued the study, 2 each from the probiotic and placebo groups, stating personal reasons (they were not interested in continuing the study and did not visit the hospital for follow-up), and 66 subjects (33 in the placebo and 33 in the *B coagulans* group) completed the study (Fig. 1). The mean age was 37.57 years, with no significant difference between the groups. The indigestion scores and other gastrointestinal symptom scores were comparable between the groups. Vital signs were measured as a part of the safety analysis, and no abnormal or out-of-range values were observed. Detailed demographics and vitals are shown in Table 1.

**Table 1:** Baseline demographics and vitals.

Parameters	*B coagulans* (N = 33)	Placebo (N = 33)	*P* value
Age (yr)	37.70 ± 13.13 (18.00, 64.00)	37.15 ± 9.81 (21.00, 57.00)	.847
Gender			
Male, N (%)	14 (42.42)	12 (36.36)	–
Female, N (%)	19 (57.58)	21 (63.64)	–
Height (cm)	159.46 ± 8.79 (145.00, 174.00)	160.81 ± 10.53 (141.00, 179.00)	.994
Weight (kg)	62.40 ± 11.85 (39.00, 84.00)	65.58 ± 9.71 (47.20, 95.10)	.237
BMI (kg/m^2^)	24.57 ± 4.66 (17.20, 35.30)	25.52 ± 4.47 (20.00, 41.20)	.401
Indigestion score (GSRS)	8.91 ± 2.50 (4.0, 14.0)	9.42 ± 2.21 (5.0, 13.0)	.38
Cumulative GSRS scores (excluding ingestion)	27.82 ± 14.48 (5.0, 48.0)	29.12 ± 13.96 (7.0, 49.0)	.71
Global patients score	3.39 ± 1.25 (1.0, 7.0)	2.88 ± 0.99 (1.0, 5.0)	.07

Mean ± SD, (Min, Max) values are given for the baseline demographics.

BMI = body mass index, GSRS = gastrointestinal symptom rating scale.

#### 3.1.1. Primary outcome parameters.

##### 3.1.1.1. GSRS gas and bloating (ingestion) subscale score

The GSRS indigestion subscale score, which includes abdominal distension, borborygmi, burping, and flatulence, changed significantly in the *B coagulans* group [8.91 ± 2.5–3.06 ± 1.98; *P* < .001; (N = 33)] when compared to placebo [9.42 ± 2.2–8.33 ± 4.22; p 0.11;(N = 33)] at the end of the study. In addition, the mean change from screening to final visit was significant in the *B coagulans* group (−5.85) in comparison to the placebo (−1.09), *P* < .001 (Table 2). A significant reduction was observed in bloating scores 3.28 ± 1.22 to 1.09 ± 0.97, *P* < .001, compared to 3.42 ± 0.97 to 2.94 ± 1.52, P = NS in placebo. The difference of −2.28 in *B coagulans* versus −0.66 in placebo was significant (*P* = .003). Similarly, the Burping (2.94 ± 1.37–1.03 ± 0.84) and Flatus (2.65 ± 1.16–0.91 ± 0.76) scores also decreased significantly in

**Table 2:** Indigestion subscale scores.

Group		Day 0	Day 15	Day 30	*P* value bet groups
Placebo (N = 33)	Bloating	3.43 ± 0.97	3.37 ± 1.39	2.94 ± 1.52	.003
	Difference	–	−0.05	−0.66	
	*P* value	–	NS	NS	
*B coagulans* MTCC 5856 (N = 33)	Bloating	3.28 ± 1.22	1.37 ± 1.00	1.09 ± 0.97	
	Difference	–	−1.97	−2.28	
	*P* value	–	<.01*	<.01*	
Placebo (N = 33)	Burping	2.77 ± 1.24	2.91 ± 1.54	2.66 ± 1.63	<.001
	Difference		0.14	−0.25	
	*P* value		NS	NS	
*B coagulans* MTCC5856 (N = 33)	Burping	2.94 ± 1.37	1.37 ± 1.00	1.03 ± 0.84	
	Difference		−1.57	−1.94	
	*P* value		<.01*	<.01*	
Placebo (N = 33)	Flatus	3.02 ± 1.29	3.31 ± 1.67	2.73 ± 1.71	<.001
	Difference		0.28	−0.45	
	*P* value		NS	NS	
*B coagulans* MTCC5856 (N = 33)	Flatus	2.65 ± 1.16	1.43 ± 1.12	0.91 ± 0.76	
	Difference		−1.23	−1.8	
	*P* value		<.01*	<.01*	
Placebo (N = 33)	Indigestion score	9.42 ± 2. 2	9.85 ± 4.06	8.33 ± 4.22	<.001
	Difference	–	0.42	−1.09	
	*P* value	–	NS	NS	
*B coagulans* MTCC5856 (N = 33)	Indigestion score	8.91 ± 2. 5	3.94 ± 2.47	3.06 ± 1.98	
	Difference	–	−4.97	−5.85	
	*P* value	–	<.001*	<.001*	

Comparison of mean indigestion subscale score of the GSRS questionnaire values between the treatment groups. Values are represented as Mean ± SD. The significance of the difference from day0 to day30 between placebo and *B coagulans* group is given in the table.

* *P* value within the groups on day15 and day 30.

*B coagulans* group. The difference of −1.97 versus −0.25 and −1.8 versus −0.45 *B coagulans* group compared to placebo was statistically significant (*P* < .001) (Table 2).

##### 3.1.1.2. Global evaluation of patients’ scores

Global evaluation of patients’ scores increased in both *B coagulans* and placebo groups at the end of the study compared to baseline. The improvement was notably superior in the

**Figure 2.**
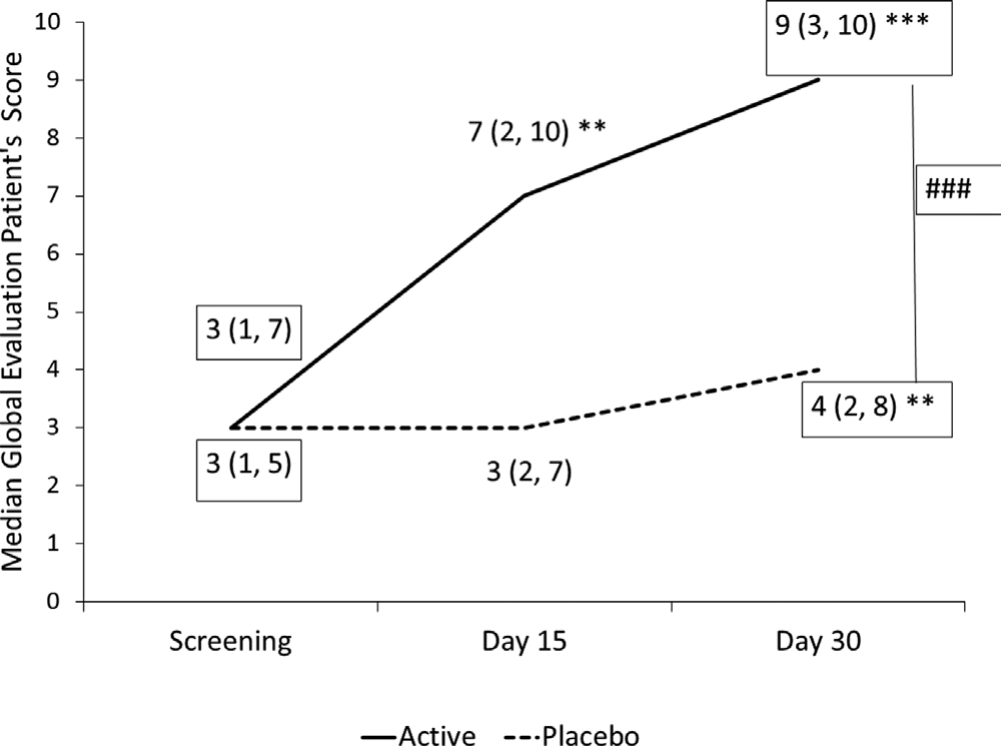
Global evaluation by patient scores; median score of global evaluation patient’s scores at screening, visit 3 (day 15) and final visit (day 30) for *B coagulans* MTCC5856) and placebo supplementation. Values are presented as Median (range) **P < .001, compared to baseline value. ^###^ *P* < .001 compared to placebo.

*B coagulans* group than in the placebo (*P* < .001). The median (range) scores changed from 3.0 (1–7) to 9.0 (3–10) (*P* < .001) in the *B coagulans* group and 3.0 (1–5) to 4.0 (2–8) (*P* < .004) in the placebo group, suggesting a placebo effect (Fig. 2).

### 3.2. Secondary outcomes

#### 3.2.1. GSRS scores (excluding indigestion subscale).

The GSRS cumulative scores for abdominal pain, reflux syndrome, diarrhea, and constipation subscales significantly improved in individuals consuming *B coagulans* MTCC 5856. The median (range) cumulative GSRS scores changed from 34.00 (5.00, 48.00) to 3.00 (1.00, 19.00) *P* < .001 in the *B coagulans* group compared to 35.00 (7.00, 49.00) to 13.00 (2.00, 55.00) *P* < .001 in the placebo group. Although the change was also significant in the placebo, the difference between the *B coagulans* and placebo was significant at the end of the study (Table 3).

**Table 3:** Cumulative GSRS questionnaire (excluding indigestion subscale).

Group		Day 0	Day 15	Day 30	*P* value between groups*
Placebo (N = 33)	Value	35.00 (7.00, 49.00)	27.00 (5.00, 63.00)	13.00 (2.00, 55.00)	<.001
	*P* value		.53	<.001	
*B coagulans* MTCC5856 (N = 33)	Value	34.00 (5.00, 48.00)	8.00 (2.00, 19.00) **	3.00 (1.00, 19.00) **	
	*P* value	-	<.001	<.001	

Comparison of cumulative GSRS Questionnaire, including abdominal pain, reflux syndrome, diarrheal, and constipation values between the treatment groups. Values are represented as Median and Range (Min, Max).

GSRS = gastrointestinal symptom rating scale.

* *P* value between the groups on day 15 and day 30.

##### 3.2.1.1. Bristol stool chart

At baseline, the Bristol stool chart ranged from type 1 to type 7 in the *B coagulans* group and type 1 to type 6 in the placebo group. Type-3 and 4, collectively representing ideal stool, were found to increase from 39.9% to 42.4% and from 24.2% to 39.39% in

*B coagulans* MTCC 5856 and placebo, respectively (Table 4). Our study did not show significant differences between probiotic and placebo groups in changing the stool type. At the end of the study, 42.2% had a normal stool, 48.8 % had a Bristol type 5 stool, and diarrhea decreased by 90% in the probiotic group. In the placebo group, participants were distributed across constipation (30.3%), normal (24.3%), type 5 (36.4%), and diarrhea (9.1%), which got redistributed across constipation (21.1%), normal (39.4%), type 5 (27.3%), and diarrhea (12.1%) with minor changes in the percentage (Table 4).

**Table 4:** Bristol stool chart score.

Parameter	*B coagulans* (N = 33)		Placebo (N = 33)	
	Screening	Final visit	Screening	Final visit
Type 1	1 (3.03 %)	0 (0.00 %)	2 (6.06 %)	0 (0.00%)
Type 2	2 (6.06 %)	2 (6.06 %)	8 (24.24 %)	7 (21.21%)
Type 3	8 (24.24 %)	1 (3.03 %)	3 (9.09%)	8 (24.24 %)
Type 4	5 (15.15%)	13 (39.39 %)	5 (15.15 %)	5 (15.15 %)
Type 5	7 (21.21%)	16 (48.48 %)	12 (36.36 %)	9 (27.27 %)
Type 6	9 (27.27 %)	1 (3.03 %)	3 (9.09%)	4 (12.12 %)
Type 7	1 (3.03 %)	0 (0.00 %)	0 (0.00 %)	0 (0.00 %)

Type 1 and Type 2 – constipation; Type 3 and Type 4 – ideal stools; Type 5 – lack of dietary fiber; Type 6 and Type 7 – diarrhoea, values are expressed as a number of subjects and percentage in parentheses.

#### 3.2.2. Safety assessment.

##### 3.2.2.1. Brain fog questionnaire

In some studies, use of probiotics has been reported to be associated with brain fog in SIBO patients. The brain fog questionnaire was included and analyzed in the study as part of the safety outcome. One subject with brain fog in the *B coagulans* group and 2 subjects in the placebo group at screening remained constant at the end of the study (See Table S1, Supplemental Digital Content, http://links.lww.com/MD/I544, which illustrates the results of brain fog questionnaire).

#### 3.2.3. Safety parameters.

One participant from the *B coagulans* group reported acidity on 2 days and nausea on 1 day during the study period. These symptoms lasted a short time and were resolved within a day (Table 5). None of the other participants reported any adverse or serious events during the study or during the 15-day follow-up period. (See Table S2, Supplemental Digital Content, http://links.lww.com/MD/I545 which illustrates the details of adverse events). The hematological (See Table S1, Supplemental Digital Content, http://links.lww.com/MD/I546- which illustrates the details of hematological assessments), hepatic, renal, biochemical parameters were in the normal range (See Table S4, Supplemental Digital Content, http://links.lww.com/MD/I547 which illustrates the details of biochemical assessments). The urine analysis was also normal and comparable to placebo (See Table S5, Supplemental Digital Content, http://links.lww.com/MD/I548, which illustrates the details of urine analysis data on safety).

**Table 5:** Summary of adverse events for all subjects.

Sub code-/group	Adverse event description	Start date	End date	SAE?	Severity	Plausible relation to study drug	Outcome
032–*B coagulans*	Acidity	October 24, 2020	October 24, 2020	No	Mild	No	Resolved
032–*B coagulans*	Vomiting	October 27, 2020	October 27, 2020	No	Mild	No	Resolved
032–*B coagulans*	Acidity	November 03, 2020	November 03, 2020	No	Mild	No	Resolved

A total of 70 subjects were enrolled and 66 subjects completed the study. One subject in *B coagulans* group reported mild adverse event which was resolved within a day.

SAE = serious adverse event.

## 4. Discussion

In this study, *B coagulans* MTCC5856 showed promising results by dramatically lowering gas, bloating, and flatus related symptoms in patients with functional bloating who did not have other serious gastrointestinal diseases. While the other GSRS ratings (reflux, diarrhea, constipation, and stomach pain) improved by 84.1% compared to 33.65% in the placebo group, the indigestion scores fell by 65.5% in the *B coagulans* MTCC5856 group compared to 11.5% in the control group. A significant improvement in the overall patient scores was observed in the probiotic group, suggesting its beneficial impact on gastrointestinal symptoms.

A previous study found that *B coagulans* GBI-30, 6086 formulations including cellulase enzyme blend improved GSRS abdominal discomfort and distension subscale scores.^[33]^ Despite not being necessary for the food cycle, the spore forming probiotic *B coagulans* is one of the potentially helpful microorganisms because it can synthesize vitamins, enzymes, proteins, and antimicrobials. It is also known for its tolerance and stability in the gastrointestinal tract.^[34]^ In the treatment of gastrointestinal illnesses such IBS, antibiotic-induced diarrhea, dysbiosis, intestinal pain, flatulence, and symptoms of gas and bloating, several *B coagulans* species have demonstrated promising therapeutic results.^[35]^

Different species of probiotics have a range of biological impacts.^[10]^ Patients with IBS have reported improvements in bloating and stomach discomfort after supplementing with *B coagulans* GBI-30, 6086,^[36]^ while those with functional bowel disorders have reported decreased constipation and abdominal pain.^[37]^ Patients with IBS who were supplemented with *B coagulans* Unique IS2 reported significant reductions in gas, bloating, abdominal pain, and satisfaction with bowel habits.^[38]^ Undiagnosed gastrointestinal discomfort, the severity of dyspepsia assessment, burping/belching, and bloating were all improved by a mixture of *B coagulans, Bacillus clausii*, and *Bacillus subtilis*.^[39]^ *B coagulans* MTCC 5856 supplementation for 90 days significantly decreased bloating, vomiting, diarrhea, abdominal discomfort, and stool frequency at the end of the research in our prior pilot study with IBS participants who had a history of diarrhea.^[30]^ The current findings compare positively with those of past studies examining the symptoms of gas and bloating linked to various gastrointestinal illnesses. The current investigation, however, was carried out on healthy volunteers.

The pathophysiology of functional abdominal bloating is not clearly understood. One of the causes of the symptom is an accumulation of gas, air, water, and fecal material in the lumen, which may result from colonic or small intestinal bacteria producing too much gas.^[40]^ Added luminal contents or gas in the colon, food intolerance, an unbalanced gut microbiota, visceral hypersensitivity, and reduced abdominal capacity are additional factors.^[41]^ SIBO is most commonly caused by colonic gram-negative aerobes and anaerobic bacterial species that can ferment carbohydrates into gas.^[42]^ Patients with functional abdominal bloating were shown to have much less gut microbial diversity, more Proteobacteria, and significantly less Actinobacteria than healthy controls.^[43]^ Additionally, these patients had a greater percentage of *Faecalibacterium*.^[43]^ Interestingly, *B coagulans* LBSC were shown to alter the gut microbiota by upregulating *Actinobacteria* and *Firmicutes* positively and down regulating *Bacteroide*tes, *Proteobacteria, Streptophyta*, and *Verrucomicrobia* in IBS patients. The study also found that people who took probiotic supplements had lower levels of *P. copri* and higher levels of *Bifidobacteria* and *F. prausnitzii*.^[44]^ Although probiotics are predicted to have strain-specific efficacy, *B coagulans* MTCC 5856 may possibly have similar effects on the composition of the gut microbiota, which can aid functional abdominal bloating and distention. When healthy patients consumed *B coagulans* MTCC 5856 instead of a placebo, we noticed minor alterations in the gut microbiome composition, with a lower proportion of Proteobacteria and a larger proportion of Actinobacteria (unpublished results). Additionally, the probiotic strain exhibits antimicrobial efficacy against both gram positive and gram negative pathogens.^[27]^ Therefore, the possible mechanism of reducing gas and bloating symptoms by *B coagulans* MTCC 5856 could be by inhibiting these gas producing microbes and positive gut microbiome modulation.

*B coagulans* MTCC 5856 at a dose of 2 billion CFU per day was well tolerated for 30 days. In the trial, there were no adverse events reported. Brain fog is a collection of transient symptoms including mental confusion, impaired judgment, poor short-term memory, and difficulty with concentration [5]. Metabolic acidosis with elevated levels of D-lactic acid in the serum was observed in patients with brain fog [6]. Carbohydrate fermentation by D-lactic producing bacteria such as *Lactobacillus* and *Bifidobacterium* in the bowel can cause lactic acidosis [7] and recent studies have reported a correlation between brain fog and probiotic use.in SIBO patients.^[45]^ In the current investigation, none of the participants had brain fog, suggesting that *B coagulans* MTCC 5856 does is not associated with lactic acidosis leading to brain fog, suggesting the safety aspect of the probiotic consumption.

The study’s limitations are the relatively smaller population size of a localized population. We conducted our study at 2 centers in South India. Further studies at multiple locations to explore the effect in different ethnicities and cohorts can be performed in a larger population. Excess gas and bloating symptoms may be associated with SIBO, as we have ruled out any other major gastrointestinal disorders in the participants. We could not perform a breath test to confirm our hypothesis as the study was carried out during the pandemic, and the ethics committee did not approve the breath test, which was also a study limitation. Future studies in a larger cohort, for a longer time, will be valuable in confirming the benefit of *Bacillus coagulans* MTCC 5856 in functional gas and bloating in the absence of other GI diseases. In addition, since our study was for 4 weeks, we still need the data on the maintenance of symptoms after cessation of the supplementation, which may also be explored in future studies in a larger population.

## 5. Conclusion

Gas and bloating symptoms are common signs of gastrointestinal disorders affecting a larger population. *Bacillus coagulans* MTCC 5856 at 2 billion CFU per day was significantly effective in alleviating the symptoms of gas and bloating in patients in the absence of other functional gastrointestinal disorders. Therefore, it can be concluded that *Bacillus coagulans* MTCC 5856 supplementation may be an effective and safe approach to reduce the symptoms of gastrointestinal symptoms in adults with abdominal gas and distension.

## Acknowledgments

We would like to thank the investigators, Dr Manjunath Patil and Dr Santosh Saklecha, and the entire clinical research team from the Sami-Sabinsa group, MS Ramaiah Medical College and Hospitals, and Santosh Hospital, Bangalore. The authors thank the statistician Mr. Kamal Kammili, M/s. Sanjeevani Bio Services Pvt. Ltd., who independently analyzed the data.

## Author contributions

**Conceptualization:** Muhammed Majeed, Kalyanam Nagabhushanam.

**Data curation:** Shaji Paulose.

**Investigation:** Lakshmi Mundkur.

**Methodology:** Kalyanam Nagabhushanam, Sivakumar Arumugam.

**Project administration:** Shaji Paulose.

**Resources:** Muhammed Majeed.

**Supervision:** Shaji Paulose.

**Validation:** Sivakumar Arumugam, Lakshmi Mundkur.

**Writing – original draft:** Lakshmi Mundkur.

**Writing – review & editing:** Muhammed Majeed, Kalyanam Nagabhushanam, Shaji Paulose, Sivakumar Arumugam, Lakshmi Mundkur.

## Supplementary Material










