# Supplementary material for: The effects of Bacillus coagulans MTCC 5856 on functional gas and bloating in adults: A randomized, double-blind, placebo-controlled study
Source: Medicine (Baltimore). 2023 Mar 3;102(9):e33109. doi: 10.1097/MD.0000000000033109 (PMC9982755; doi:10.1097/MD.0000000000033109)
Supplement: Supplementary file 1 [file medi-102-e33109-s001.pdf]

Table S1. Brain Fog questionnaire score

PARAMETER	*B. coagulans* (N=33)	PLACEBO (N=33)
**Screening visit**		
Yes	1 (3.03 %)	2 (6.06 %)
No	32 (96.97 %)	31 (93.94 %)
**Final visit**		
Yes	1 (3.03 %)	2 (6.06 %)
No	32 (96.97 %)	31 (93.94 %)

Brain Fog Questionnaire Score percentage was recorded on screening and final visit.
