# Supplementary material for: The effects of Bacillus coagulans MTCC 5856 on functional gas and bloating in adults: A randomized, double-blind, placebo-controlled study
Source: Medicine (Baltimore). 2023 Mar 3;102(9):e33109. doi: 10.1097/MD.0000000000033109 (PMC9982755; doi:10.1097/MD.0000000000033109)
Supplement: Supplementary file 2 [file medi-102-e33109-s002.pdf]

Supplementary Results

Table S2. Display of Adverse Events for one participant.

CATEGORY	*B. coagulans* Group (N=33)	PLACEBO Group (N=33)	Overall
**All Participant Subjects**	35	35	70
**Number of subjects with at least one AE**	00	01	01
**Subject Discontinuation due to AE**	00	00	00
**Subject with SAE or Death**	00	00	00

Only one subject reported the adverse event in *B. coagulans* group. A list of adverse events experienced by study subjects in active group are provided.
