# Supplementary material for: The effects of Bacillus coagulans MTCC 5856 on functional gas and bloating in adults: A randomized, double-blind, placebo-controlled study
Source: Medicine (Baltimore). 2023 Mar 3;102(9):e33109. doi: 10.1097/MD.0000000000033109 (PMC9982755; doi:10.1097/MD.0000000000033109)
Supplement: Supplementary file 4 [file medi-102-e33109-s004.pdf]

Supplementary Results

Table S4. Biochemical parameters.

**PARAMETER**	***B. coagulans* (N=33)**		**Placebo (N=33)**	
	**Screening**	**Final Visit**	**Screening**	**Final Visit**
**Total Bilirubin(mg/dL)**	0.67 ±0.28	0.67 ±0.25	0.73 ± 0.41	0.80 ± 0.35
**Alkaline Phosphatase (U/L)**	76.28 ±31.32	84.78 ±15.67	74.95 ±18.87	84.16 ±18.90
**AST (IU/L)**	19.42 ±5.30	20.30 ± 5.47	20.29 ± 9.40	21.83 ± 4.79
**AST (IU/L)**	18.99 ±7.78	21.61± 7.43	24.45 ± 16.49	24.32 ± 10.11
**eGFR**	93.42±22.52	91.20 ± 21.73	91.20±27.32	84.69 ± 25.22
**Urea (mg/dL)**	21.23± 9.28	20.31± 7.32	22.43 ± 5.70	21.32± 5.95
**Uric Acid (mg/dL)**	4.11 ± 1.47	30.71(3.07)	4.35 ±1.20	31.45 (3.21)
**Serum Creatinine (mg/dL)**	1.79±4.81	0.82±0.16*	0.81±0.19	0.87±0.17
**Sodium (mmol/L)**	132.84 ± 23.27	138.67 ± 2.67	137.06 ± 2.41	138.94 ± 3.21
**Potassium (mmol/L)**	4.40±0.45	4.33±0.40	4.70±1.78	4.37±0.43
**Chloride (mmol/L)**	100.49±2.56	100.52±2.76	100.27±2.34	101.98±3.08

Data represents the Mean± SD. None of the parameters showed any significant difference from baseline to end of the study in *B. coagulans* and the placebo groups.
