# Supplementary material for: The effects of Bacillus coagulans MTCC 5856 on functional gas and bloating in adults: A randomized, double-blind, placebo-controlled study
Source: Medicine (Baltimore). 2023 Mar 3;102(9):e33109. doi: 10.1097/MD.0000000000033109 (PMC9982755; doi:10.1097/MD.0000000000033109)
Supplement: Supplementary file 5 [file medi-102-e33109-s005.pdf]

Supplementary Results

Table S5 Urine analysis

PARAMETER	*B. coagulans* (N=33)		Placebo (N=33)	
	**Screening**	**Final Visit**	**Screening**	**Final Visit**
**Urine Color**				
Slightly Yellow	2 (6.06 %)	3 (9.09 %)	26 (78.79 %)	3 (9.09 %)
Pale Yellow	28 (84.85 %)	22 (66.67 %)	6 (18.18 %)	21 (53.53 %)
Straw	3 (9.09%)	4 (12.12 %)	0 (0.00 %)	5 (15.15 %)
Yellow	0 (0.00 %)	4 (12.12 %)	91.20±27.32	4 (12.12 %)
**Urine Transparency**				
Clear	12 (36.36%)	21 (65.71 %)	11(33.33 %)	27(81.82 %)
Slightly Turbid	18 (54.54%)	11 (33.33 %)	19 (57.57%)	4 (12.12 %)
Turbid	3 (9.09%)	1 (3.03 %)	3 (9.09 %)	2 (6.06 %)
**Specific Gravity**	1.02 ±0.01	1.02 ±0.01	1.02 ±0.01	1.02 ±0.01
**Protein**				
Absent	33 (100.00 %)		33 (100.00 %)	
Present	0 (0.00 %)		0 (0.00 %)	
**Ketone bodies**				
Absent	33 (100.00 %)		33 (100.00 %)	
Present	0 (0.00 %)		0 (0.00 %)	

Data represents the Number of participants (%). None of the parameters showed any significant difference from baseline to end of the study in *B. coagulans* and the placebo groups.
